# Nonlabor Income and Age at Marriage: Evidence From China’s Heating Policy

**DOI:** 10.1007/s13524-018-0732-1

**Published:** 2018-11-27

**Authors:** Junhong Chu, Haoming Liu, I. P. L. Png

**Affiliations:** 1 NUS Business School, National University of Singapore, 15 Kent Ridge Drive, Singapore, 119245 Singapore; 2 Department of Economics, National University of Singapore, 1 Arts Link, Singapore, 117570 Singapore

**Keywords:** Age at marriage, Regression discontinuity, Nonlabor income, China

## Abstract

We exploit China’s heating policy to investigate how nonlabor income affects marriage. From the mid-1950s, the policy gave substantial subsidies to urban residents north of the Huai River. Applying geographic regression discontinuity, we find that with the policy, urban men in the north married 15 months earlier than men in the south. The difference is substantial given that the average age at first marriage is 24.9 years for urban men in the south. The effect is larger for later birth cohorts, which is consistent with the progressive implementation of the policy. The effect is smaller among women, consistent with women having less power in the household than men. There is no effect among rural residents, who did not benefit from the heating policy.

**Electronic supplementary material:**

The online version of this article (10.1007/s13524-018-0732-1) contains supplementary material, which is available to authorized users.

## Introduction

Between 1982 and 2010, the worldwide age at first marriage rose from 22.4 to 24.7 among women and from 25.1 to 26.5 among men, narrowing the spousal age gap by 0.9 years (World Bank n.d.). The age at marriage has important economic and social implications. First and foremost, because most children are born after marriage, the age at marriage—particularly the woman’s—affects fertility (Baizán et al. 2003; Cecos et al. 1982; Oppenheimer et al. 1997). Second, the age at marriage might affect marriage stability, which in turn affects children’s welfare (Bloom and Reddy 1986; Bongaarts 1978; Lehrer 2008; Oppenheimer 1988; Rotz 2016; Thornton and Rodgers 1987). Third, the age at marriage might affect investment in education, especially among women (Field and Ambrus 2008; Iyigun and Lafortune 2016), with consequent effects on productivity, fertility, and child development (Bhrolcháin and Éva 2012; Isen and Stevenson 2011). Fourth, the spousal age gap might affect wages. To the extent that younger women marry older men, specialization within a family affects women’s wages relatively more (Loughran and Zissimopoulos 2009). Fifth, because women tend to marry older men but live longer, a smaller spousal age gap would benefit women by reducing the widowhood length.

Owing to the economic and social importance of marriage, policy-makers and scholars are keen to understand the effect of income and wealth on marriage (Nakosteen and Zimmer 1987; Schneider 2011; Weiss et al. 2018; Yu and Xie 2015). However, empirical studies are challenging because of possible endogeneity. For instance, earnings affect the timing of marriage, but marriage also affects the growth of earnings. The most robust empirical tests have exploited exogenous shocks that raised the earnings of the mainly male workers in the oil and gas industry, finding mixed results. The 1979 oil shock raised the marriage rate of young men in U.S. oil-producing areas (Jelnov 2016). By contrast, in the late 1990s and 2000s, increases in U.S. oil and gas production resulting from new fracking technology did not cause more men to marry (Kearney and Wilson 2018).

Theoretically, higher wages might either increase or reduce the gains from marriage and thus would affect the age at marriage in conflicting ways. In the Becker (1973, 1974) model of the gains from marriage due to specialization in outside *vis-à-vis* domestic work, an increase in wages raises income (increasing the gains from marriage) and also raises the time cost of domestic work (reducing the gains from marriage). Moreover, higher earnings may be associated with more inequality in earnings, in which case, higher-earning men might defer marriage to prove their earnings potential. With greater inequality of earnings among men, the gains from searching for husbands would be higher; as a result, women would search longer, thus delaying marriage (Bergstrom and Bagnoli 1993; Cherlin et al. 2016; Gould and Paserman 2003; Loughran 2002).

By contrast, an increase in *nonlabor income* raises income but does not affect the time cost of domestic work, thereby unambiguously raising the gains from marriage. Here, we extend the Becker (1973, 1974) model and show that higher nonlabor income directly increases the gains from marriage by raising men’s earnings and women’s home production. Assuming that men’s wages are higher than women’s, marriage leads men to work more in the labor market, while women work more at home. Because men substitute their hours of housework with their wife’s time, they benefit more from marriage. Nevertheless, women might share some of the gains from higher nonlabor income through bargaining with their husbands.

We test the theoretical predictions by exploiting an exogenous increase in nonlabor income resulting from Chinese government policy.^1^ In the mid-1950s, the government of China decided to subsidize heating during winter. The subsidy took the form of cash allowances, amounting to 6 % to 12 % of winter monthly salary, coupled with the distribution of free or subsidized coal. To limit the cost of the heating policy, the government restricted the subsidy to urban residents north of a boundary, demarcated by the Huai River in the east and the Qin Mountains in the west. The government did not provide allowances to or subsidize heating for rural residents or those who lived south of the Huai-Qin boundary.

The arbitrary division of urban China into areas with and without subsidized heating provides a unique quasi-natural experiment to investigate the causal effect of nonlabor income on the age at marriage. Importantly, because the heating subsidies were the same for all urban residents of a county, it would not have affected search for marital partners. We apply geographical regression discontinuity (GRD) analysis—a method cited by Duncan (2008) for demography research and applied by Legewie (2013)—to identify the effect of the heating policy on the age of first marriage among urban residents in the three provinces spanned by the Huai River: Henan, Anhui, and Jiangsu.

The concept (and assumption) of GRD is that people living just north of the Huai River are essentially comparable with those living just south of the river, except for the heating policy. Then, any north-south difference in the age at marriage at the Huai River is attributable to the heating policy.^2^ Applying a sharp GRD design, we find that among urban men, the age at first marriage was 1.25 years (15 months) lower in the north than the south. This difference is meaningful given that the average age at first marriage is 24.9 years in the south. Among female urban residents, the age at first marriage was lower in the north than south (by 7.8 months) only in the 1956–1965 birth cohort. These results are robust to the GRD method and bandwidth, and inclusion of control variables.

Our interpretation of the north-south difference as being due to the heating policy is buttressed by three falsification exercises. First, applying GRD analysis to urban men by birth cohort, we find no significant north-south difference among the 1926–1935 birth cohort (most of whom married before the heating policy) or the 1936–1945 cohort (some of whom married before the heating policy), but a significant difference only in the 1946–1955 and 1956–1965 cohorts. Second, we do not find any significant north-south difference among rural men, which is consistent with the heating policy benefiting only urban residents. Third, we also find no significant north-south difference in the age at first marriage with counterfactual boundaries (latitude 32.5 degrees north, or the Huai River plus or minus 50 km).

The weaker north-south difference in the age at first marriage among women suggests that they benefited less than men from the gains from marriage due to the heating policy. This disparity is consistent with men enjoying more power in the household than women, social norms that men bear more of the financial responsibilities of marriage than women, and norms that associate men’s status with their earnings.

Our findings have implications for government subsidies for health, housing, heating, poverty alleviation, and other purposes that increase nonlabor income. Besides their intended objectives, these subsidies encourage earlier marriage by increasing nonlabor income. Further, to the extent that the effect on the age at marriage is stronger for men than women, it reduces the spousal age gap and shortens the widowhood period for women and increases their well-being. In the specific context of China’s heating policy, our findings suggest that the policy possibly yields countervailing benefits that should be weighed against the costs, such as the harmful effects of emissions on health and mortality (Almond et al. 2009; Chen et al. 2013; Xiao et al. 2015).

## Model

Following Becker (1973), we assume individuals consume only a domestic good, *Z*, which is produced at home using inputs purchased from the market, *x*, and time, *t*. For simplicity, specify the production function of the domestic good as Cobb-Douglas,

$$ Z={x}^{\upalpha}{t}^{1-\upalpha}, $$

where α is the share of market inputs with their price normalized to 1. Given that the marginal utility of the domestic good is always positive, the individual seeks to

1 $$ \underset{x,t}{\max }Z\ \mathrm{subject}\ \mathrm{to}\ x=v+ wl, $$

where *v* is nonlabor income, *w* is the wage rate, and *l* is the quantity of labor supplied in the labor market, with *t* + *l* = 1. For a single person, the optimal solution is

2 $$ {Z}_i^{\ast }={\upalpha}^{\upalpha}{\left[1-\upalpha \right]}^{1-\upalpha}{S}_i{w}_i^{\upalpha -1}, $$

where *S*_*i*_ = *v*_*i*_ + *w*_*i*_ represents the individual’s total income. Subscripts *i* = *f*, *m* denote the spouse, respectively, with lower and higher wage rate, *w*_*m*_ *> w*_*f*_. (See the online appendix, section A, for the proofs of the optima for a single person and a married couple.)

For a married couple, any reduction in the domestic good would render someone worse off without making anyone better off. Hence, they will maximize the joint production of the domestic good. Assume that the time of husband and wife is perfectly substitutable in home production and that the wife continues to work in the labor market after marriage. Then the husband should allocate all his time to market work, *l*_*m*_ = 1. If *l*_*m*_ *<* 1, he should work more in the labor market, and she should work less, which would raise their earnings and production of the domestic good. Accordingly, for a married couple with the wife working in the labor market, the household production is

3 $$ Z={x}^{\upalpha}{t}_f^{1-\upalpha}, $$

where *x* = *v*_*m*_ + *v*_*f*_ + *w*_*m*_ + *w*_*f*_ [1 *− t*_*f*_ ]. The optimal solution is

4 $$ {Z}^{\ast }={\upalpha}^{\upalpha}{\left[1-\upalpha \right]}^{1-\upalpha}\left[{S}_m+{S}_f\right]{w}_f^{\upalpha -1}, $$

where *S*_*m*_ and *S*_*f*_ are as defined earlier. The difference in the production of the domestic good between marriage and singlehood is

5 $$ G={Z}^{\ast }-{Z}_m^{\ast }-{Z}_f^{\ast }={\upalpha}^{\upalpha}{\left[1-\upalpha \right]}^{1-\upalpha}{S}_m{w}_m^{\upalpha -1}\left[1-{r}^{\upalpha -1}\right], $$

where *r* = *w*_*m*_ / *w*_*f*_ is the ratio of the husband’s to wife’s wages.

The assumption *w*_*m*_ *> w*_*f*_ implies that *r >* 1. Further, α < 1, and hence, *G >* 0: that is, marriage increases the production of the domestic good. Accordingly, people will prefer to marry rather than remain single. The gains from marriage do not vary with the woman’s nonlabor income but increase with the man’s nonlabor income. Formally, with respect to the woman’s nonlabor income,

6 $$ \frac{\partial G}{\partial {v}_f}=0,\mathrm{and}\ \frac{\partial^2G}{\partial {v}_f^2}=0. $$

With respect to the man’s nonlabor income,

7 $$ \frac{\partial G}{\partial {v}_m}={\upalpha}^{\upalpha}{\left[1-\upalpha \right]}^{1-\upalpha}{w}_m^{\upalpha -1}\left[r-1\right]>0,\mathrm{and}\ \frac{\partial^2G}{\partial {v}_m^2}=0. $$

Intuitively, with constant returns to scale in home production, the gains from marriage are driven purely by specialization. Specifically, the gains are the number of hours that the man spent on home production when single multiplied by the husband-wife wage differential. Given the wage rates, higher nonlabor income implies longer working hours at home as a single and hence benefit more from marriage.

Referring to Eq. (7), the second derivative suggests that the marginal gain from marriage for men does not depend on their nonlabor income. However, if a household’s utility is concave in *Z*, then the gains from marriage would be lower for men with higher earnings. In our empirical analysis, we proxy earnings by education and test its moderating effect on the effect of nonlabor income on timing of marriage.

By contrast, as long as women’s wages are lower than men’s, married women will always spend time on home production. The increase in the woman’s hours in home production after marriage depends only on her husband’s pre-marriage hours in home production; the woman’s hours in home production do not vary with her own nonlabor income. Hence, the gains from marriage do not vary with women’s nonlabor income.

Our theory implies that an increase in nonlabor income would raise the gains from marriage by raising men’s earnings and women’s home production. However, our theory is silent on the division of the gains from marriage. To the extent that men have more power in the household than women, men bear more of the financial responsibilities of marriage than women, or if men and women share the gains from marriage according to their earnings, the gains would be larger for men than women. Intuitively, a large gain encourages people to marry early. Hence, our model implies that an increase in nonlabor income reduces the age at first marriage. The impact is likely to be larger for men if they enjoy a larger share of the gains from the rise in nonlabor income. We test these predictions in the upcoming section, Robustness.

## Institutional Context

China’s heating policy originated in the first five-year plan (1953–1957). The long-term intent was to provide central heating. In the interim, pending the construction of central heating systems, the government decided to pay cash allowances and subsidize coal purchase (State Council of China 1956). To economize on resources, the government limited the heating policy to urban residents living north of a boundary marked by the Huai River in the east and the Qin Mountains in the west (State Council of China 1956). The Huai-Qin boundary runs through the provinces of Jiangsu, Anhui, Henan, Shaanxi, and Gansu. The heating policy did not apply to rural residents or those living south of the Huai-Qin boundary.

The Chinese government built the first central heating system only in 1958 in Beijing and subsequently expanded it to other urban areas. However, even in the year 2000, only 27 % of Beijing had central heating. The coverage elsewhere was very likely lower. Consequently, for most people, the heating policy took the form of cash allowances and free or subsidized coal for home use.

The heating policy was a major welfare benefit for urban residents living north of the Huai-Qin boundary. Table 1 lists the cash allowances during winter months stipulated by the central government in Anhui, Henan, and Jiangsu (State Council of China 1978). During the sample period, most urban residents were employed directly or indirectly by the government (Xie et al. 2009). Work units typically gave allowances in excess of the amounts stipulated by the central government. Based on our surveys of residents of the three provinces, the cash allowance ranged from 6.7 % to 11.5 % of monthly winter income. Besides the cash allowance, the heating policy also provided free or subsidized heating coal (refer to the online appendix Table A1, panel B).

**Table 1:** Heating policy: Implementation

		Henan	Anhui	Jiangsu
a.	Earliest Implementation	1957	1979	1957
b.	Average Salary of Urban Worker (1978) (yuan per month)	49.17	46.25	42.75
c.	Minimum Heating Allowance (1978/1979) (yuan per month)	4.00	3.00	3.00
d.	Minimum Heating Allowance Relative to Average Salary (1978/1979) (%)	8.14	6.49	7.02
e.	Heating Allowance Relative to Monthly Salary (1960–1999) (%)	11.50	6.73	9.57
		(3.16)	(3.11)	(6.66)

*Sources:* Row a: China State Council (1956) and documents listed in online appendix Table A1. Row b: China Compendium of Statistics 1949–2008 (*Xin zhongguo 60 nian tongji ziliao huibian*). Row c: China State Administration of Labor and Ministry of Finance (1978). Row d: row b divided by row c. Row e: Based on authors’ survey of urban residents of Henan, Anhui, and Jiangsu, with each cell reporting mean and standard error (shown in parentheses).

Although the government promulgated the heating policy in 1957, some evidence suggests that it was implemented progressively. For instance, an Anhui provincial government document states that the heating cash allowance was implemented only in 1979. However, the central government specified the cash allowance in Anhui as 9 yuan per year in 1978 (State Council of China 1978), and respondents in our surveys reported receiving the allowance in the 1970s. Importantly, the cash allowances and free or subsidized coal were lump-sum payments and not related to work income.

Another important feature of the Chinese institutional context is the *hukou* (household registration) system established in 1958, by which individuals have legal status only in their registered place of residence. This affects birth, marriage, divorce, housing, food and other rationed items, education, and employment. In particular, only people with the local *hukou* qualify for the heating subsidy. If two persons with different *hukou* registrations marry, it may take years to reregister the spouse’s *hukou*. Therefore, it is reasonable to assume that people could not migrate to take advantage of heating subsidies and that, indeed, any person who left their registered place would lose their heating subsidy for some years. Based on the 2000 census, we calculated that more than 90 % of urban residents lived in their birth provinces.

The third relevant feature of the Chinese institutional context is labor markets. Until the economic reforms beginning in 1978, almost all urban Chinese residents were government employees, either directly or indirectly (Xie et al. 2009). Surprisingly, wages varied considerably even under Communism. Table A1 (panel A) in the online appendix presents monthly wage rates for workers in various industries in Henan, Anhui, and Jiangsu provinces. Wages varied by responsibility, skill, and occupational hazard. For instance, in the mining industry, underground workers were paid more than above-ground workers. Presumably, workers who put in longer hours and more effort could increase their prospects for promotion to better-paid positions. Moreover, workers could earn additional wages for overtime work and rewards for producing beyond their quota. Some workers were paid hourly or received a per piece rate and thus would earn more by working longer.

To check whether labor markets and marriage conditions during the study period conformed with the assumptions of our theory, we surveyed 51 female and 44 male urban residents of Henan, Anhui, and Jiangsu who married before 1980. As Table 2 reports, nearly 40 % of women and one-half of the men reported that before marriage, the husband earned more than the wife. Both women and men considered the spouse’s income and job to be important in marriage. Approximately 20 % of women and more than 40 % of men reported that they had delayed marriage because of low income. More than 10 % of women and more than 40 % of men reported that if they had received a windfall gain, they would have married earlier.

**Table 2:** Marriage: Factors and behavior

	Women	Men	Difference
Age at Marriage			*t* Test
Mean	23.9	24.8	–0.83
SD	3.18	3.46	(*p* = .22)
Who Earned More Before Marriage			Rank-Sum Test
Self	5	27	3.74**
Neither	25	3	(*p* < .001)
Spouse	21	14	
Importance to Marriage			Chi-Squared Test
Spouse’s income important	34	21	3.48^†^
Spouse’s income not important	17	23	(*p* = .06)
Spouse’s job important	41	27	4.20*
Spouse’s job not important	10	17	(*p* = .04)
Timing of Marriage			Chi-Squared Test
Delayed due to low income	11	19	5.11*
Not delayed	40	25	(*p* = .02)
Would marry earlier if getting cash windfall	6	19	12.02**
Would not marry earlier	45	25	(*p* = .001)
After Marriage			Chi-Squared Test
Spent more time on housework	47	25	16.08**
Did not spend more time on housework	4	19	(*p* < .001)
Worked harder	12	23	8.39**
Did not work harder	39	21	(*p* = .004)
Worked more overtime	6	19	12.02**
Did not work more overtime	45	25	(*p* = .001)
Number of Observations	51	44	

*Notes:* The sample is restricted to people with urban *hukou* at the time of marriage and who married before 1980. The rightmost column reports statistics and *p* values of tests of differences between women and men.

*Source:* Authors’ survey of urban residents in Henan, Anhui, and Jiangsu provinces conducted in August 2017.

^†^*p* < .10; *p < .05; **p < .01

After marriage, more than 90 % of women reported doing more housework. By contrast, more than 50 % and more than 40 % of men reported working harder and more overtime after marriage. Broadly, the survey findings fit the assumptions of our theory that men earned more than women before marriage, income was a factor in marriage, and nonlabor income enlarged the gains from marriage by enabling men to increase paid work in the labor market while women increased domestic work.

## Empirical Strategy

To investigate the effect of nonlabor income on marriage, consider the following linear reduced-form model:

8 $$ {a}_{ji}={\mathbf{X}}_{ji}^{\prime}\upbeta +\gamma {y}_{ji}+{v}_{ji}, $$

where *a*_*ji*_ represents the age at first marriage of individual *j* with urban *hukou* living in county *i*; **X**_*ji*_ is a vector of personal and community characteristics that affect the decision to marry, with β as their coefficient; *y*_*ji*_ is nonlabor income, with γ as the coefficient; and *ν*_*ji*_ is a random error term.

The ordinary least squares (OLS) estimate of γ might be biased if *ν* is correlated with *y*. One obvious source for such a correlation is earnings, which affects both age at marriage and nonlabor income. Moreover, people with higher nonlabor income might also share other characteristics, such as social status, that affect their attractiveness in marriage.

The limitation of the heating policy to urban residents living north of the Huai-Qin boundary provides a natural quasi-experiment. Given the sharp change in policy along the boundary, it is intuitive to apply GRD analysis. Duncan (2008) cited RD as a method for causal inference in demography research, and Legewie (2013) used RD to study U.S. immigration.

Assuming that subjects are continuously distributed around the boundary and that all factors besides the heating policy that affect the age at first marriage vary smoothly around the boundary, the GRD method produces a consistent estimate of the coefficient of nonlabor income. Almond et al. (2009), Chen et al. (2013), Wang (2014), and Wang and Hong (2015) applied GRD around the Huai-Qin boundary to investigate the effect of emissions on respiratory illnesses and the effect of rice culture on economic liberalization and income. To apply GRD, let nonlabor income be

9 $$ {y}_{ji}={\mathbf{Z}}_{ji}^{\prime}\upphi +\updelta {N}_i+{\upvarepsilon}_{ji}, $$

where **Z**_*ji*_ is a vector of observed personal and community characteristics, with ϕ as their coefficient; *N*_*i*_ indicates county *i* being situated north of the boundary, with δ as the coefficient; and ε_*ji*_ is random error. The north indicator, *N*_*i*_, represents the heating policy. Substituting Eq. (9) in Eq. (8), the reduced-form model resolves to

10 $$ {a}_{ji}={\mathbf{X}}_{ji}^{\prime}\upbeta +{\mathbf{Z}}_{ji}^{\prime}\upphi \upgamma +\updelta \upgamma {N}_i+{\upeta}_{ji}, $$

where η_*ji*_ = *v*_*ji*_ + ε_*ji*_.

Further, define *d*_*i*_ to be the distance of county *i* from the boundary, with *d*_*i*_ *>* 0 and *d*_*i*_ *<* 0 for counties north and south of the boundary, respectively. Let *f* (*·*) and *g*(*·*), with *g*(*d*_*i*_) = 0 for *d*_*i*_ *<* 0, be smooth functions that represent other factors (besides the heating policy) that affect nonlabor income and are correlated with distance. We then replace Eq. (10) with the GRD model of the age at first marriage as a function of the heating policy:

11 $$ {a}_{ji}=\upmu {N}_i+f\left({d}_i\right)+g\left({d}_i\right)+{\upeta}_{ji}, $$

where the coefficient of the heating policy is μ ≡ δγ. The heating policy raises nonlabor income, δ *>* 0; thus, μ and γ have the same sign. A negative μ implies that nonlabor income reduces the age at first marriage. Because the functions *f*(*·*) and *g*(*·*) may differ, the GRD approach allows the effect of the other factors to differ between north and south.

The key identifying assumption of the GRD analysis is that all factors other than the heating policy vary continuously at the boundary. Under this assumption, η_*ji*_ is a random error, and Eq. (11) provides a consistent estimate of μ. In particular, we do not need to control for personal and community characteristics, **X**_*ji*_ and **Z**_*ji*_. Obviously, if the sample is limited to people who live very close to the boundary, then the estimate would not be sensitive to whether we control for these characteristics. With a larger sample that extends further from the boundary, the north-south differences in these characteristics might be larger. However, assuming that **X**_*ji*_ and **Z**_*ji*_ change continuously with distance from the boundary, then functions *f*(*·*) and *g*(*·*) should account for their impact on the dependent variable. By the same reasoning, not controlling for unobserved characteristics that are correlated with *y*_*ji*_ and *a*_*ji*_ in Eq. (11) would not bias the estimate of μ. Nevertheless, including personal and community characteristics reduces the residual variation in the age at first marriage, and thereby improves the precision of the estimates. In robustness checks, we compare the estimates of μ with different sets of control variables.

There are two ways to apply regression discontinuity (Lee and Lemieux 2010). The parametric approach specifies *f*(*·*) and *g*(*·*) as polynomial functions and estimates Eq. (11) by OLS using all observations. The nonparametric approach estimates Eq. (11) over an optimally selected subset of the data. Intuitively, as the sample is narrowed to counties closer to the boundary, the assumptions on the functional form become less restrictive. In the nonparametric approach, the selection of the bandwidth (i.e., choosing which counties to include in the analysis) is important. Here, we focus on the mean squared error (MSE)–optimal method (Calonico et al. forthcoming) with symmetric bandwidth and stipulate locally quadratic distance functions. In robustness checks, we use the parametric and the nonparametric approach with asymmetric bandwidth.^3^

**Fig. 1:**
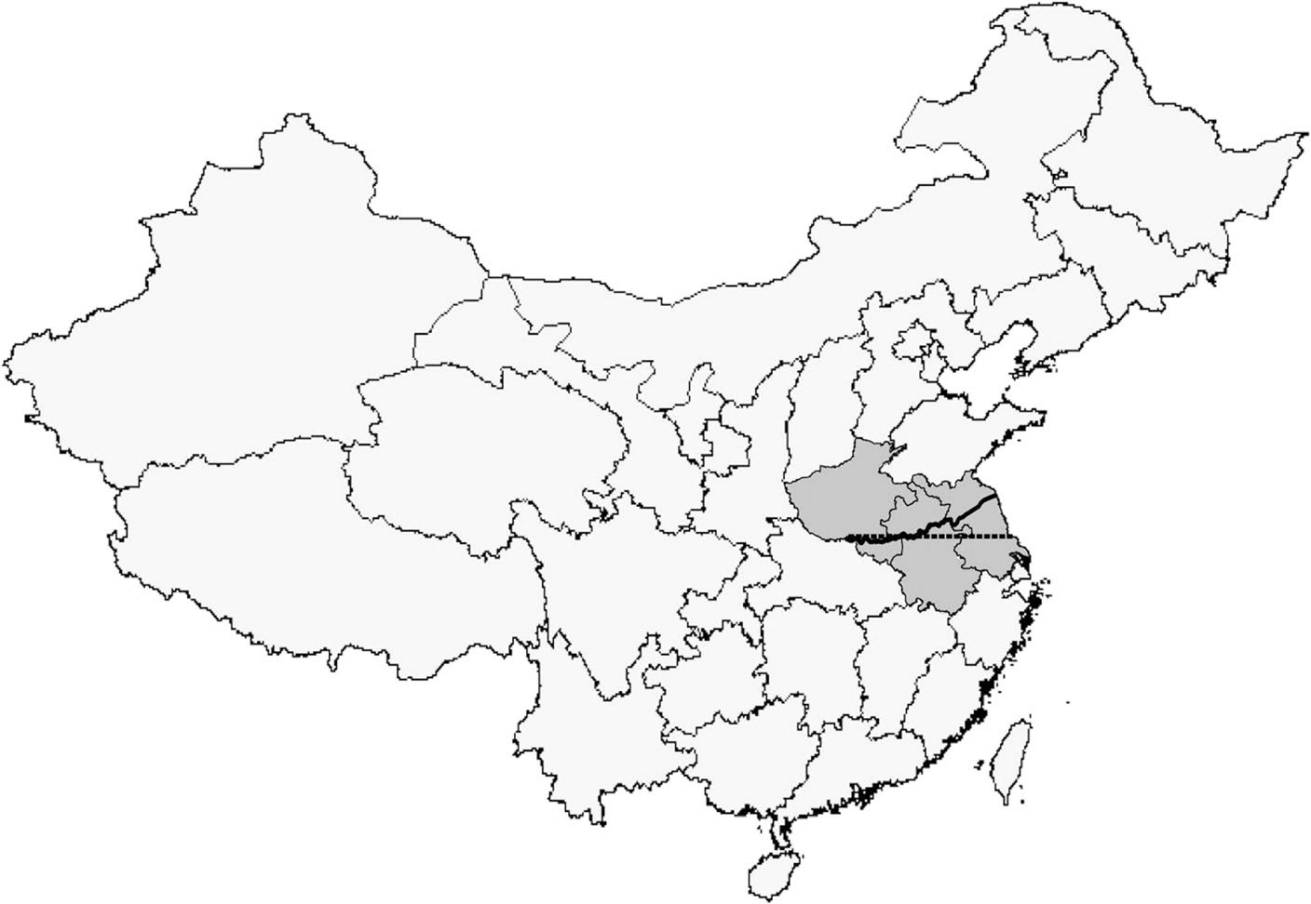
Heating policy: Huai River. From east to west, the boundary of the heating policy follows the Huai River (solid line). To the west of Huai River, the boundary follows the Qinling Mountains, which are sparsely populated. Thus, we focus on the Huai River part of the heating policy boundary, which runs through Jiangsu (right), Anhui (center), and Henan (left). The 32.5 degree north latitude is represented by the dashed line

In applying GRD around the Huai-Qin boundary, one concern is that the Qin Mountains are thinly populated, which challenges the GRD assumption that subjects are continuously distributed around the discontinuity. By contrast, the Huai River basin in Henan, Anhui, and Jiangsu is an unobstructed geographical area, with easy movement throughout. Importantly, areas immediately north and south of the river are well-populated. Accordingly, to meaningfully apply GRD analysis, we limit our study to these three provinces (Fig. 1).

## Data

Administratively, China is divided into provinces, which are further divided into prefectures, which are further divided into counties. We carry out our analyses by county because the heating policy is administered as such (State Council of China 1978). Henan, Anhui, and Jiangsu provinces comprise 47 prefectures made up of 166 counties located south and 206 counties north of the Huai River.

China’s 2000 Population Census is the first census to report age at first marriage. From the 1 % sample of the census, we collect data on age at first marriage, current marital status, family, ethnicity, education, and employment for individuals born 1925–1965. The marriages of those born before 1925 might have been affected by the Sino-Japanese War, World War II, or the Chinese Civil War, and relatively few survived until 2000. Accordingly, we exclude people born before 1925 from the study. Obviously, information on age at marriage is available only for those who marry. Among those with urban *hukou* and born before 1965, 98.8 % of men and 99.6 % of women had married by the year 2000. By comparison, among urban residents born between 1966 and 1975, the marriage rate is only 79 % for men and 91 % for women. Accordingly, we limit our study to people born before 1965.

Our main analyses focus on individuals with urban *hukou*. In falsification analyses, we also consider rural residents. To minimize the impact of possible migration (notwithstanding the *hukou* policy), we limit our analysis to individuals who resided in the same neighborhood since birth. Our final sample comprises 72,264 individuals in 372 counties, of whom 46 % resided north of the Huai (Tables 3 and 4).

**Table 3:** Summary statistics: Marriage data

	Age at First Marriage		Marriage Rate	
	Women	Men	Women	Men
North	22.63	23.58	99.70	99.23
	(2.95)	(3.29)	(5.50)	(8.76)
	[11,507]	[21,423]	[11,542]	[21,590]
South	22.86	24.88	99.55	98.38
	(3.04)	(3.40)	(6.70)	(12.61)
	[16,353]	[22,338]	[16,427]	[22,705]
Total	22.77	22.92	99.61	98.79
	(3.01)	(4.20)	(6.23)	(10.91)
	[27,860]	[43,861]	[27,969]	[44,295]
North-South Difference	–0.23	–1.30	0.15	0.84
*t* Statistic	–6.34	–40.65	1.95	8.13

*Notes:* The table reports means, standard deviations in parentheses, and frequencies in brackets. The *t* statistic is for the difference between north and south. The sample comprises residents in the 2000 census in Henan, Anhui, and Jiangsu who were born in 1926–1965, had an urban *hukou*, and did not move from neighborhood of birth.

**Table 4:** Summary statistics: Individual and county characteristics

Variables	Unit	*N*	Mean	SD	Min.	Max.
Age at First Marriage	Years	71,621	23.67	3.34	16	45
Early Marriage	Indicator	72,264	0.65	0.48	0	1
Male	Indicator	72,264	0.61	0.49	0	1
Han Chinese	Indicator	72,264	0.98	0.13	0	1
Age in Year 2000	Years	72,264	48.59	10.51	35	74
Years of Schooling	Years	72,264	8.74	3.36	0	18
Parental Years of Schooling	Years	6,830	4.26	3.78	0	16
Government Official/Professional	Indicator	72,264	0.18	0.39	0	1
Multigenerational Household	Indicator	71,621	0.27	0.44	0	1
Confucian Temples Density	Per million persons	372	1.01	0.95	0	3.22
Rice Culture	Proportion	372	0.33	0.31	0	0.91
Gender Ratio	Proportion	372	1.18	0.20	0.57	2
SOE Employment	Proportion	372	0.95	0.03	0.88	0.999
GDP per Capita	‘000 yuan	372	9.46	8.03	1.68	37.74
North	Indicator	72,264	0.46	0.50	0	1
Jiangsu	Indicator	72,264	0.49	0.50	0	1
Henan	Indicator	72,264	0.30	0.46	0	1
Anhui	Indicator	72,264	0.21	0.41	0	1
Distance to Huai River	Kilometers	372	167.15	103.91	0.49	407.91
Average Daily Winter Temperature	°C	372	2.24	1.46	–1.81	5.03
Average Winter Precipitation	Millimeters/day	372	0.78	0.49	0.18	2.64
Average Winter Sunshine Hours	Hours/day	372	5.06	0.41	3.97	6.20
Relative Cohort Loss Rate		371	–0.44	0.20	–1.04	0.33
Education Loss of Sent Down Youth	Years	372	–0.04	0.09	–0.40	0.14
Historical Agricultural Population	Proportion	224	0.59	0.24	0.05	0.96
Historical Tenancy Rate	Proportion	342	0.78	0.13	0.21	0.995

*Notes:* The sample comprises residents in the 2000 census in Henan, Anhui, and Jiangsu who were born in 1926–1965, had an urban *hukou*, and did not move from neighborhood of birth.

We divide the sample into four 10-year birth cohorts: 1926–1935, 1936–1945, 1946–1955, and 1956–1965. The marriages of people in these cohorts might have been disrupted by three upheavals: the Chinese Civil War (1946–1949), the Great Famine (1959–1961), and the Cultural Revolution (1966–1976).

In the 1926–1935 cohort, 82 % had married by 1958; thus, their decisions to marry were largely unaffected by the heating policy but might have been affected by the Civil War. In the 1936–1945 cohort, 10 % married before 1958, and another 18 % married during the Great Famine (1959–1961). Their decisions to marry might have been affected by the heating policy and the famine. In the 1946–1955 cohort, all married after 1961, more than one-half married during the Cultural Revolution, and approximately one-third married afterward. Their decisions to marry might have been affected by the heating policy and the Cultural Revolution. In the 1956–1965 cohort, 96 % were married in 1978 or after. The marriage decisions in the north should have been fully affected by the heating policy and not much affected by the Cultural Revolution.

Table 3 summarizes the marriage data. Marriage is almost universal at 99.6 % among women and 98.8% among men. We calculate the age at first marriage as the year of first marriage minus birth year. Among urban residents, women’s average age at first marriage is 22.6 in the north and 22.9 in the south; comparable figures for men are 23.6 in the north and 24.9 in the south. Apparently, urban northerners married earlier than urban southerners, and the difference is more pronounced among men than women.

Table 4 summarizes other individual and county characteristics that might affect marriage. Almost all the people were Han Chinese, the average person was born in 1951 and had 8.7 years of schooling, and 18 % were employed by the government or in a professional occupation.

Marriage might be affected by social norms and culture, which might differ north and south of the Huai River. We account for norms and culture in several ways. First, we use an indicator (multigeneration household) of individuals living with either their parents or married adult children. People in multigenerational households are likely to be more family-oriented or less-affluent (Goldscheider and Lawton 1998). Second, we consider Confucianism, a system of philosophical, ethical, and sociopolitical thinking that emphasizes family and obedience to authority. Confucianism might be stronger in the north given that it originated in Shandong Province, which abuts Henan, Anhui, and Jiangsu to the north (Kung and Ma 2014). Third, Talhelm et al. (2014) theorized that growing rice requires collective action, making societies that historically grew rice more collectivist. Single people in collectivist cultures might marry earlier. Rice-growing requires a temperate climate, and thus rice culture would vary from north to south.

Two other factors that affect marriage that might differ across the Huai River are gender balance and economic development. For each county, we construct gender ratio as the number of males per female among people born in 1926–1965. We represent economic development by the proportion of employment in state-owned enterprises (SOEs) and gross domestic product (GDP) per capita.

Finally, using Google Maps, we plot the geocoordinates and calculate the shortest distance from each county seat to the Huai River. From the China Meteorological Administration, we collect daily instrumental observations of minimum and maximum temperatures at 64 stations over the years 1956–1987. We match each county seat to the nearest weather station. The average daily winter temperature (December to February) is 1.27°C and 3.44°C in the north and south, respectively.

We assume that prefecture-level data (density of Confucian temples, rice culture, SOE employment, and GDP) apply to all counties within the prefecture. This might result in classical measurement error; if so, the estimated coefficients would be biased downward.^4^

## Estimates

The Chinese government promulgated the heating policy with effect from 1957. Our model predicts that the heating policy raised the gains from marriage, particularly for men. Accordingly, we analyze men and women separately. Further, because the heating policy might have been implemented progressively and thus might have had a greater effect on the marriage of people born later, we analyze the effect of the policy by birth cohort.

**Fig. 2:**
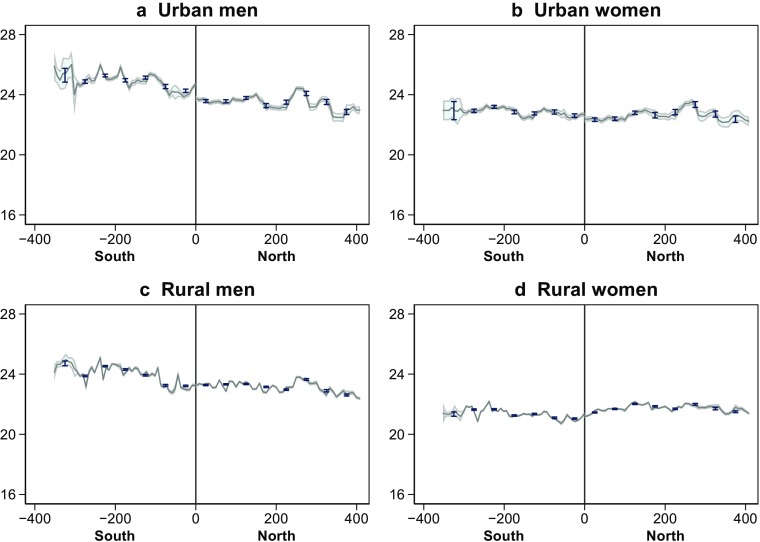
Age at first marriage by *hukou* and gender. Sample comprises all birth cohorts (born 1926–1965). Graphs depict fitted values from local polynomial estimate of the individual age at first marriage on distance from the Huai River, and corresponding 95 % confidence intervals. Dots represent average age at first marriage within 50 km bins

Panels a–d in Fig. 2 plot the county average age at first marriage among men and women with urban and rural *hukou* by distance from the Huai River. The figure also depicts the fitted values from local polynomial regressions of the individual age at first marriage on the distance from the Huai, along with the corresponding 95 % confidence intervals. The graphs evince a discrete north-south difference in the age at first marriage among urban men as well as urban women, but not among rural men and rural women. In these regressions, the unit of analysis is an individual person, making the confidence intervals very tight.

**Fig. 3:**
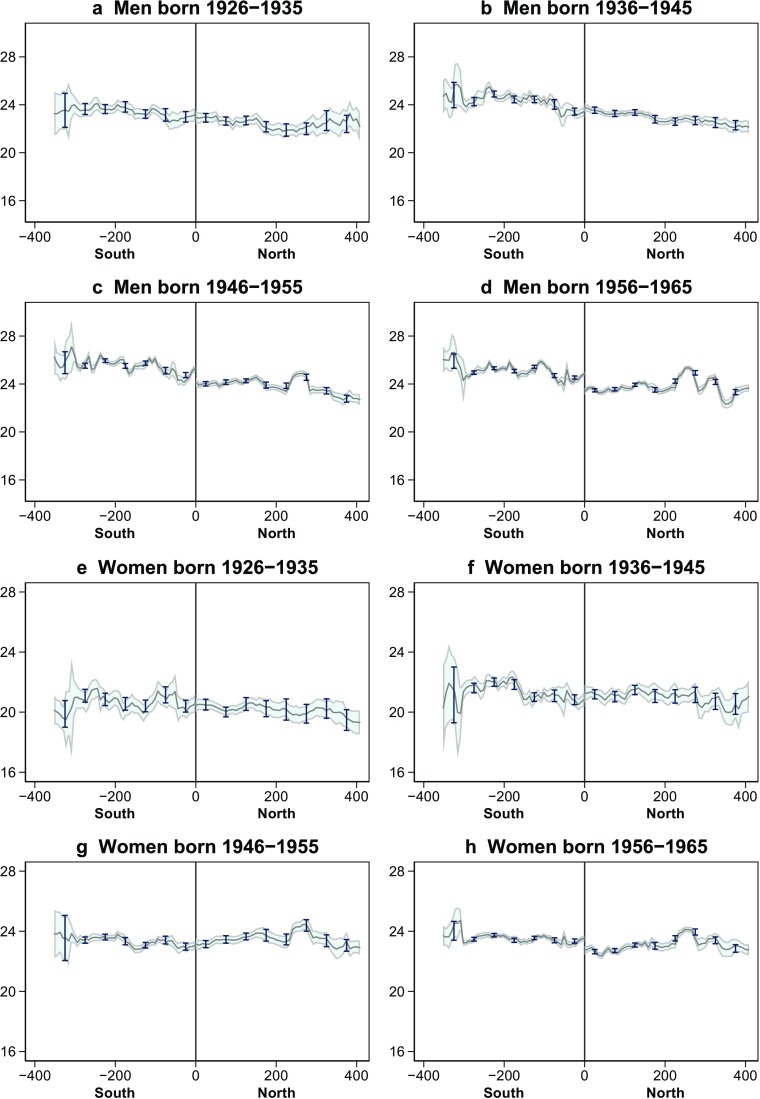
Age at first marriage by birth cohort (urban residents). Samples comprise all male and female residents with urban *hukou* in the respective birth cohorts. Graphs depict fitted values from local polynomial estimate of the individual age at first marriage on distance from the Huai River, and corresponding 95 % confidence intervals. Dots represent average age at first marriage within 50 km bins

Next, to explore differences by the age of people when the heating policy came into effect, panels a–h of Fig. 3 plot the fitted local polynomial regressions and corresponding 95 % confidence intervals. For comparison, these figures also plot the average age at first marriage within 50 km bins. The graphs for men show no north-south difference in the oldest and next-to-oldest cohorts, but they do show discrete north-south differences in the later cohorts. The graphs for women evince a north-south difference only in the youngest cohort.

Following up on the graphical analysis, Table 5 presents GRD analyses of the age at first marriage among urban men and women. Panel A shows a significant north-south difference in the age at first marriage of –1.25 years (SE = 0.43) among all urban men, which is large given the average age at first marriage of 24.9 years in the south. The north-south difference is not significant in the two older cohorts but is significant in the two younger cohorts. In the youngest cohort, northerners married about 1.5 years (18 months) earlier than southerners. Panel B reports the estimates for urban women, showing a significant north-south difference in the age at first marriage only in the youngest cohort. The estimate suggests that in the youngest cohort, northerners married 0.65 years (7.8 months) earlier than southerners, which is large given the average age at first marriage of 23.5 years in the youngest cohort in the south.

**Table 5:** Urban residents: Regression discontinuity

	All Cohorts	Age at First Marriage				Married Early
		1926–1935	1936–1945	1946–1955	1956–1965	1956–1965
Variables	(1)	(2)	(3)	(5)	(6)	(7)
A. Men						
North	–1.25**	0.07	0.58	–1.20*	–1.49**	0.18**
	(0.43)	(0.48)	(0.43)	(0.49)	(0.40)	(0.05)
Number of observations	11,260	2,893	4,599	4,141	4,895	5,026
Counties	86	194	193	111	90	91
BIC	59,270	16,462	25,352	21,941	23,668	5,845
Bandwidth (km)	78.96	169.42	167.64	101.49	82.46	84.87
B. Women						
North	–0.15	0.51	0.79^†^	0.18	–0.65*	0.09*
	(0.27)	(0.48)	(0.42)	(0.28)	(0.29)	(0.04)
Number of observations	13,820	1,274	1,893	3,952	6,124	9,433
Counties	157	148	139	159	144	174
BIC	70,003	6,620	9,855	20,318	28,502	11,646
Bandwidth (km)	136.53	131.47	119.53	137.33	125.27	145.26

*Notes:* Coefficients were estimated by OLS with quadratic distance polynomials, rectangular kernel functions, and MSE-optimal bandwidth. The sample comprises married residents in 2000 census in Henan, Anhui, and Jiangsu who were born in 1926–1965, had an urban *hukou*, and did not move from neighborhood of birth. The dependent variable is age at first marriage for columns 1–6, and fraction of residents married early (before age 25 for men and before age 23 for women). Standard errors, clustered by county, are shown in parentheses.

^†^*p* < .10; *p < .05; **p < .01

Table 5 shows that the mean age at marriage was lower among individuals who benefited from the heating policy than among others. Was this effect due to more people marrying early or fewer people marrying late? To investigate, we conduct a GRD analysis on the probability of marriage before the government-promoted age of late marriage, 25 for men and 23 for women.^5^ Column 6 in Table 5 reports the estimates for the youngest cohort. Both northern men and northern women were more likely to marry before the age of late marriage. Apparently, the heating policy did encourage people to marry earlier.

We find an economically and statistically significant north-south difference in the age at first marriage and interpret it as being due to the heating policy. This interpretation is buttressed by three sets of falsification exercises. First, as shown in panel A of Table 5, the north-south difference is not significant in the two older cohorts, which were not affected by the heating policy. Second, as shown in Table A3 of the online appendix (column 2), there is no significant north-south difference in the age at first marriage among men with rural *hukou*, which is consistent with the heating policy benefiting only urban residents.

Third, we experiment with counterfactual boundaries for the heating policy. Previous GRD studies of China’s heating policy defined the boundary by latitude 32 or 32.5 degrees north (Almond et al. 2009; Chen et al. 2013; Wang 2014) rather than the actual geocoordinates of the river. Referring to Fig. 1, the Huai River follows latitude 32.5 degrees north quite closely in Henan but not in Anhui and Jiangsu. This discrepancy means that people on both sides of the latitude would be eligible for heating allowances and subsidies, challenging the GRD method. As shown in columns 3–5 of Table A3 (online appendix), in GRD estimates with the boundary stipulated as latitude 32.5 degrees north, or 50 km north or south of the Huai River, the coefficient of north is positive and not statistically significant.^6^

### Robustness

Discontinuities at the Huai River in other factors that affect marriage represent a potential threat to our identification strategy. Focusing on urban men born in 1956–1965, we first check graphically whether other factors that possibly affect marriage are discontinuous at the Huai River. These factors are ethnicity, age, education, occupation, social norms and culture, the availability of marriage partners (gender ratio), employment in state-owned enterprises, and overall economic development.

Online appendix Fig. A1 shows geographical discontinuities at the Huai River in government/professional occupation and Confucianism, and, less precisely, age and rice culture. The figure suggests that people living north of the Huai were more likely to be employed in government or professional occupations. Subject to the proviso that the information on occupation is from the 2000 census, and not at the time of marriage, the difference in occupation might challenge our identification strategy. The discontinuity in Confucianism may be an artifact of the data being at the prefectural level and the coarseness giving the appearance of a discontinuity. Nevertheless, this discontinuity does threaten our identification strategy to the extent that Confucianists tend to marry earlier.

Figure A1 (online appendix) suggests that people living north of the Huai tend to be older. Older cohorts would have married earlier, and the apparent discontinuity therefore does challenge our identification strategy. The difference in rice culture would confound our identification strategy to the extent that rice-growing societies are more collectivist (Talhelm et al. 2014) and encourage earlier marriage. However, rice culture is weaker in the north, which suggests that our estimates are conservative.

To check whether our results are sensitive to the exclusion of other factors that possibly affect marriage, we show in columns 2–9 of Table 6 nonparametric GRD estimates including each of these factors individually and all of them together as additional controls. Some of these other factors do significantly affect the age at marriage. Yet, in each robustness check, the coefficient of north is quite similar to the preferred estimate without additional controls and precisely estimated. These results suggest that the smooth functions of distance to the Huai River effectively control for the effect of other factors.

**Table 6:** Urban men (born 1956–1965): Confounding variables

	Preferred Estimate	Individual Covariates	Family Orientation	Confucianism	Rice Culture	Gender Ratio	SOE Employment	Economic Development	All Controls
Variables	(1)	(2)	(3)	(4)	(5)	(6)	(7)	(8)	(9)
North	–1.49**	–1.38**	–1.38**	–1.40**	–1.42**	–1.24**	–1.25**	–1.23**	–1.12**
	(0.40)	(0.36)	(0.36)	(0.36)	(0.35)	(0.35)	(0.29)	(0.31)	(0.27)
Han Chinese		–0.81**	–0.81**	–0.78*	–0.80*	–0.81**	–0.87**	–0.74**	–0.76**
		(0.31)	(0.31)	(0.31)	(0.30)	(0.30)	(0.29)	(0.28)	(0.28)
Age in Year 2000		0.16**	0.16**	0.16**	0.16**	0.16**	0.16**	0.16**	0.16**
		(0.01)	(0.01)	(0.01)	(0.01)	(0.01)	(0.01)	(0.01)	(0.01)
Years of Schooling		0.11**	0.11**	0.11**	0.11**	0.11**	0.11**	0.11**	0.11**
		(0.02)	(0.02)	(0.02)	(0.02)	(0.02)	(0.02)	(0.02)	(0.02)
Parental Years of Schooling		0.08**	0.08**	0.08**	0.08**	0.08**	0.08**	0.08**	0.08**
		(0.02)	(0.02)	(0.02)	(0.02)	(0.02)	(0.02)	(0.02)	(0.02)
Missing Parental Years of Schooling		–0.14	–0.20	–0.13	–0.14	–0.14	–0.12	–0.09	–0.12
		(0.14)	(0.25)	(0.14)	(0.14)	(0.14)	(0.15)	(0.14)	(0.25)
Government Official or Professional		–0.30**	–0.30**	–0.29**	–0.29**	–0.27**	–0.26*	–0.23*	–0.20*
		(0.10)	(0.10)	(0.10)	(0.10)	(0.10)	(0.10)	(0.10)	(0.10)
Multigenerational Household			–0.07						–0.04
			(0.20)						(0.20)
Confucian Temples				0.15**					0.07
				(0.07)					(0.07)
Rice Culture					–0.71				–0.33
					(0.71)				(0.47)
Gender Ratio						–1.20**			–0.83*
						(0.44)			(0.36)
SOE Employment							0.10**		0.05
							(0.03)		(0.03)
GDP per Capita								0.12**	0.10**
								(0.03)	(0.02)
Province Fixed Effects	Yes	Yes	Yes	Yes	Yes	Yes	Yes	Yes	Yes
Number of Observations	4,895	4,895	4,895	4,895	4,895	4,895	4,895	4,895	4,895
Counties	90	90	90	90	90	90	90	90	90
BIC	23,668	23,540	23,538	23,537	23,544	23,547	23,467	23,459	23,473
Bandwidth (km)	82.46	82.46	82.46	82.46	82.46	82.46	82.46	82.46	82.46

*Notes:* The coefficients were estimated by OLS with quadratic distance polynomial and MSE-optimal bandwidth. The sample comprises married male residents in the 2000 census born in 1956–1965, with urban *hukou* in 90 counties within bandwidth of 82.46 km (as in preferred estimate from Table 5, panel A, column 6), who did not move from neighborhood of birth. The dependent variable is age at first marriage. All estimates control for province fixed effects. Standard errors, clustered by county, are shown in parentheses. Column 1: preferred estimate from Table 5, panel A, column 6. Column 2: controlling for indicator of Han Chinese, age, years of schooling and parental years of schooling, and indicator for government or professional occupation. Column 3: also controlling for indicator for couples living with parents or married children. Column 4: also controlling for ratio of number of Confucian temples in Ming and Qing dynasties in prefecture to population (Kung and Ma 2014). Column 5: also controlling for percentage of sown land devoted to rice paddies in the early 1990s. Column 6: also controlling for county gender ratio (number of men to 100 women). Column 7: also controlling for proportion of SOE employment. Column 8: also controlling for county GDP per capita. Column 9: all controls.

^†^*p* < .10; *p < .05; **p < .01

The coefficients of these control variables should be interpreted with caution because the variables might be correlated with unobserved factors. For instance, Confucianism emphasizes obedience to authority as well as family. Government policy urged people to marry later, while family-oriented people might marry earlier. The positive coefficient of Confucianism suggests that the former effect dominates. Similarly, even though people who voluntarily live in multigenerational households might be more family-oriented (and hence, marry earlier), those forced to live in multigenerational households due to lack of housing might have difficulty finding a spouse (hence, might marry late).

To further check the robustness of the empirical findings, section B of the online appendix reports additional GRD estimates. One set (Table A3, columns 6–9) checks sensitivity to method and applies the nonparametric approach with linear distance functions, alternative kernel functions, and multilevel model (Stata routine *meglm*). Another set checks sensitivity to sample. Columns 10–11 in Table A3 report estimates that apply the nonparametric approach with the sample limited to urban residents, and that include seven adjacent provinces: Shanxi, Hebei, and Shandong to the north, and Hubei, Jiangxi, Zhejiang, and Shanghai to the south. The estimate in column l of Table A3, applies the parametric approach to the 10 provinces. Another set of robustness checks (Table A4, panels A and B) applies the parametric GRD approach to the entire sample. Another set of estimates (Table A5, panels C and D) applies the nonparametric approach with asymmetric bandwidths (Calonico et al. forthcoming). Our finding that the age at first marriage among urban residents is significantly lower in the north, particularly among men in later cohorts, is robust to these alternative methods and sample.^7^

### Mechanism

Our interpretation of the north-south difference in the age at first marriage as being due to the effect of the heating policy on nonlabor income is supported by the differential and much stronger effect on men than on women. This difference is consistent with men enjoying more power in the household than women (Mangyo 2008; Shu et al. 2013), social norms that men bear more of the financial responsibilities of marriage than women, or norms that men’s status is positively correlated with their earnings.

Next, we investigate the moderating effect of income on the effect of the heating policy. To the extent that the heating policy affects the gains from marriage through nonlabor income and the marginal utility of the home-produced good diminishes with quantity, the heating policy should have less effect on higher-earning people. Lacking direct information on individual income, we use two proxies: years of education and employment by the government or in a professional occupation.

Better-educated people would qualify for more responsible positions and earn higher wages (online appendix Table A1), and education would not change much between the time of marriage and the 2000 census. As column 1 of Table 7 reports, the coefficient of the interaction between north and education is positive, which is consistent with diminishing marginal utility of the home-produced good. However, the estimate is imprecise, perhaps owing to insufficient variation in the education within the sample. Table A6 in the online appendix reports estimates on the entire sample of counties, not limited to the optimal bandwidth. The coefficient of north is negative, significant, and smaller among more-educated individuals (with more than a high school education) than the less-educated. In an estimate including the interaction between north and education, the coefficient of the interaction is positive, significant, and similar to the estimate in column 1 of Table 7.

**Table 7:** Urban men (born 1956–1965): Mechanism and alternative explanations

	Education	Government Official/Professional	Winter Climate			Great Famine	Send Down	Cultural Revolution	
			Temperature	Precipitation	Sunshine			Agriculture	Tenancy
Variables	(1)	(2)	(3)	(4)	(5)	(6)	(7)	(8)	(9)
North	–1.50**	–1.53**	–1.44**	–1.40**	–1.42**	–1.47**	–1.23**	–1.21**	–1.45**
	(0.40)	(0.39)	(0.19)	(0.19)	(0.19)	(0.40)	(0.37)	(0.31)	(0.36)
Moderator	0.05**	–0.17	0.18^†^	–0.00	–0.47*	0.45	–4.53*	–0.01**	–2.19*
	(0.02)	(0.14)	(0.10)	(0.42)	(0.20)	(0.73)	(1.74)	(0.00)	(0.88)
North × Moderator	0.05	0.21	–0.25*	–2.65**	0.45^†^	–1.18	–7.14*	0.02**	5.07**
	(0.03)	(0.19)	(0.12)	(0.68)	(0.24)	(0.89)	(3.12)	(0.01)	(1.35)
Number of Observations	4,895	4,895	4,895	4,895	4,895	4,895	4,895	3,542	4,712
Counties	90	90	90	90	90	90	90	63	85
Weather Stations			26	26	26				
BIC	23,654	23,683	23,680	23,660	23,680	23,681	23,628	17,226	22,817
Bandwidth (km)	82.46	82.46	82.46	82.46	82.46	82.46	82.46	82.46	82.46

*Notes:* Coefficients were estimated by OLS with quadratic distance polynomial and MSE-optimal bandwidth. The sample comprises married male residents in the 2000 census born in 1956–1965, with urban *hukou* in 90 counties within bandwidth of 82.46 km (as in baseline estimate from Table 5, panel A, column 6), who did not move from neighborhood of birth. The dependent variable is age at first marriage. Standard errors, clustered by county (columns 1–2) and by weather station (columns 3–5), are shown in parentheses. Column 1: effect of heating policy contingent on years of education. Column 2: effect of heating policy contingent on indicator for government official or professional occupation. Columns 3–6: effect of heating policy contingent on winter climate (daily average temperature, precipitation, and sunshine hours in December, January, and February during 1956–1987 specified as difference from mean), with standard errors adjusted for small number of clusters using Stata routine *clustse*. Columns 6–8: effect of heating policy contingent on intensity of the Great Famine (relative cohort loss rate), the Send Down movement (loss of education among sent-down youth), and Cultural Revolution (historical agricultural population and tenancy rate) specified as difference from mean.

^†^*p* < .10; *p < .05; **p < .01

In pre-reform China, government employees and professionals earned more in wages and benefits than others. As column 2 of Table 7 reports, the coefficient of the interaction between north and government/professional occupation is positive, which is consistent with diminishing marginal utility of the home-produced good. However, the estimate is imprecise, perhaps because of measurement error. The estimate uses the occupation at the census and thus depends on the occupation remaining unchanged since marriage. Table A6 in the online appendix reports estimates for the entire sample of counties. The coefficient of north is negative, significant, and smaller among those in government/professional occupations relative to others.

Subject to the imprecision of the estimates, we infer some evidence that the heating policy had less effect on people with higher incomes. This interpretation is consistent with diminishing marginal utility of the home-produced good in our model of the effect of nonlabor income on the gains from marriage.

### Alternative Explanations

We show that the heating policy was associated with earlier marriage and interpret this association as the effect of an increase in nonlabor income on the gains from marriage. Yet, because our study is not a controlled experiment, the effect of the heating policy is open to alternative explanations.

One alternative explanation relates to income inequality. If people care about absolute income inequality, the policy should not have affected search behavior because it did not affect the dispersion of income. Nevertheless, the policy did reduce proportionate inequality, which might have affected search. To the extent that men bear greater financial responsibility, it should have a larger impact on women’s marriage searches. However, we find a relatively larger effect on men, suggesting that changes in inequality do not explain the relation between the heating policy and age at marriage.

Another set of explanations is that wealth defines eligibility for marriage; savings buffer against future uncertainty and stress (Schneider 2011); or more prosaically, higher nonlabor income helps to pay for the costs of marriage, such as home, furnishings, and ceremonies. These alternative explanations do not account for the differential effect of the heating policy on men compared with women. However, we cannot definitely rule out these explanations if combined with a social norm that the husband bears more of the financial responsibilities of marriage.

The heating policy might also affect marriage directly through thermal comfort. The policy makes being together more comfortable (hence, encouraging people to marry earlier). By this theory, the heating policy should have more of an effect in areas where the climate is more severe. Columns 3–5 of Table 7 report estimates contingent on measures of winter climate. The coefficient of north interacted with winter temperature is negative, implying that where the winter is milder, the effect of the heating policy was larger. This result is inconsistent with the thermal comfort theory. The coefficient of north interacted with precipitation is negative and significant, which is consistent with the thermal comfort theory. The coefficient of north interacted with sunshine hours is positive but imprecise. Nevertheless, even if thermal comfort does explain the interaction between the heating policy and winter climate, the estimated north-south differences in columns 3–5 of Table 7 are close to the baseline estimate. This finding suggests that the heating policy did affect marriage beyond thermal comfort.

Another possible set of explanations relates to the Great Famine (1959–1961) and the Cultural Revolution (1966–1976), which overlapped with the timing of the progressive implementation of the heating policy. Although both shocks certainly disrupted marriage, the issue is whether they differentially affected marriage north and south of the Huai River. Column 6 of Table 7 reports an estimate contingent on the severity of the Great Famine, as represented by the dip in county level population during the famine period (Chu et al. 2016). Columns 7–9 of Table 7 report estimates contingent on the severity of the Cultural Revolution, as represented by the county-level average loss of education due to urban youths being sent down to the countryside, and two historical measures of the strength of Communist Party in the county (Xu et al. 2018). The estimated coefficient of north is robust to all these contingencies.

## Discussion

Through geographical regression discontinuity analyses, we find a significant north-south difference around the Huai River in the age of first marriage among men with urban *hukou* born between 1946–1965 and women with urban *hukou* born between 1956–1965. We interpret these differences as being due to the Chinese government’s heating policy, which provided cash allowances and subsidized coal to urban residents north of the Huai River. The results are consistent with the implications of our extended Becker (1973, 1974) model, which shows that an increase in nonlabor income raises the gains from marriage by raising men’s labor supply while reducing women’s.

Lacking information on the individual cash allowance or coal subsidy, we can only roughly calculate the elasticity of the age at first marriage with respect to nonlabor income. This calculation is biased upward given that it ignores the coal subsidy. According to our survey, the heating allowance amounted to 6.7 % to 11.5% of respondents’ monthly salaries. Further, we estimate that the age at first marriage among urban men in the north was 1.25 years lower, which is 5.3 % lower than the average of 23.6 years. Hence, a 1 % increase in nonlabor income was associated with roughly a 0.46 % to 0.79 % reduction in the age at marriage.

Our findings bear implications for government policies that raise nonlabor income. Such policies increase the gains from marriage and thus encourage and accelerate marriage. With regard to China’s heating policy, our findings point to additional benefits (besides thermal comfort) to balance against the deleterious effects on health. The age at marriage affects fertility, stability of marriage, and education—effects that are particularly consequential for countries trying to encourage marriage and fertility. Further, the spousal age gap possibly affects wages and widowhood. Given the difficulty of identifying the causal effect of the age at marriage on these outcomes, China’s heating policy might provide a useful identification strategy.

A clear limitation of our empirical design is that the heating policy raised the nonlabor income of both men and women. Hence, for women, we cannot distinguish the direct effect of the increase in nonlabor income (which, according to our theory, is zero) from the indirect effect through men sharing their gains from marriage. An obvious direction for future work is to investigate the effect of an increase in women’s nonlabor income separately from an increase in men’s nonlabor income.

## Electronic Supplementary Material

ESM 1 (PDF 541 kb)

## Footnotes

1 The gains from marriage also manifest in the rate of marriage (Choo and Siow 2006). However, marriage was almost universal in China during our study period. Accordingly, we use age at marriage to represent the gains from marriage.

2 As we explain in the upcoming section, Empirical Strategy, the GRD accounts for differences between people in other factors that affect the age at marriage by functions (which may differ between north and south) of the distance from the river.

3 Calonico et al. (forthcoming) generalized the nonparametric methods developed by Ludwig and Miller (2007), Imbens and Kalyanaraman (2011), and Calonico et al. (2014).

4 See online appendix Table A2 for details regarding the data sources and construction of the variables.

5 From 1982, the government promoted late marriage by awarding 7 to 12 days of additional paid leave to women and men who married after ages 23 and 25, respectively (Ministry of Labor and Ministry of Finance 1980).

6 See section B of the online appendix for more falsification tests.

7 Section C of the online appendix reports similar results from a difference-in-differences (DID) analysis.
