# Supplementary material for: Nonlabor Income and Age at Marriage: Evidence From China’s Heating Policy
Source: Demography. 2018 Nov 27;55(6):2345–70. doi: 10.1007/s13524-018-0732-1 (PMC6290655; doi:10.1007/s13524-018-0732-1)
Supplement: Supplementary file 1 — (PDF 541 kb) [file 13524_2018_732_MOESM1_ESM.pdf]

Appendix A: Proofs

Single person

The single person maximizes

$$Z = x^{\alpha} t^{1-\alpha}, \tag{A-1}$$

subject to $x = v + wl$ and $t + l = 1$. Substituting from the constraints in (A-1), the maximand simplifies to

$$Z = \Big[v + w[1-t]\Big]^{\alpha} t^{1-\alpha}. \tag{A-2}$$

The first-order condition with respect to t,

$$-w\alpha\Big[v + w[1-t]\Big]^{\alpha-1} t^{1-\alpha} + [1-\alpha]\Big[v + w[1-t]\Big]^{\alpha} t^{-\alpha} = 0.$$

Simplifying,

$$-w\alpha\Big[v + w[1-t]\Big]^{-1} t + [1-\alpha] = 0,$$

and so,

$$t = \frac{[1-\alpha][v+w]}{w}.$$

Substituting in (A-2),

$$\begin{aligned} Z &= [v + w - wt]^{\alpha} \left[\frac{[1-\alpha][v+w]}{w}\right]^{1-\alpha} \\ &= \Big[\alpha[v+w]\Big]^{\alpha} \left[\frac{[1-\alpha][v+w]}{w}\right]^{1-\alpha} \\ &= \alpha^{\alpha}[1-\alpha]^{1-\alpha}[v+w]w^{\alpha-1}. \end{aligned} \tag{A-3}$$

Substituting $S = v + w$ yields (1).

Married couple

The husband works full time in the labor market and let the wife work $1 - t_f$ in the labor market. Then they will purchase the quantity of inputs, $x = v_m + v_f + w_m + w_f[1 - t_f]$.

Accordingly, their joint home product will be

$$Z = [S_m + S_f - w_f t_f]^{\alpha} t_f^{1-\alpha}. \tag{A-4}$$

The first-order condition with respect to t_f is

$$-\alpha w_f [S_m + S_f - w_f t_f]^{\alpha-1} t_f^{1-\alpha} + [1-\alpha][S_m + S_f - w_f t_f]^{\alpha} t_f^{-\alpha} = 0,$$

which simplies to

$$-\alpha w_f [S_m + S_f - w_f t_f]^{-1} t_f + [1-\alpha] = 0,$$

or

$$\alpha w_f t_f = [1-\alpha][S_m + S_f - w_f t_f],$$

which implies that

$$t_f = \frac{[1-\alpha][S_m + S_f]}{w_f}. \tag{A-5}$$

Substituting in (A-4),

$$\begin{aligned} Z &= \Big[S_m + S_f - [1-\alpha][S_m + S_f]\Big]^{\alpha} \left[\frac{[1-\alpha][S_m + S_f]}{w_f}\right]^{1-\alpha} \\ &= \alpha^{\alpha} [1-\alpha]^{1-\alpha} [S_m + S_f] {w_f}^{\alpha-1}, \end{aligned}$$

which is (3).

Appendix B. Data and Supplementary Estimates

To further check whether our estimated north/south difference in age of first marriage is driven by factors than are not related to heating policy, we run another 20 counterfactual analyses by setting hypothetical policy boundaries at 50 km intervals from 500 kms south to 500 kms north of the Huai River. Figure A2 plots these hypothetical boundaries. The figure shows that while the Huai River is in the middle of a flat region (the Huang-huai and Jiang-huai plains), most of the hypothetical policy boundaries cross mountains as well as plains. Obviously, mountainous areas are thinly populated, and so, the bulk of the people on the two sides of a hypothetical boundary crossing mountains could live hundreds of kms apart. Hence, people living on different sides of the boundary could behave quite differently, which a smooth function of distance would not properly control for. This violates a fundamental assumption of GRD, and the GRD estimate is likely to be sensitive to the choice of the boundary.

The estimates using hypothetical boundaries are reported in Table A4 and plotted in Figure A3. Most of the coefficients are not significantly different from zero. The coefficients that are statistically significant are unstable. For instance, among the three clusters of coefficients that are significant - between 450 and 250 kms south, between 150 and 200 kms north, and between 350 and 450 kms north, the estimates oscillate between positive and negative. The instability of these estimates could be due to the hypothetical boundaries crossing mountains. The only robust estimate is that for the actual Huai River boundary. These estimates provide further support to our conclusion that the heating policy indeed affected the age of first marriage of the affected cohorts.

Figure A1. Possible confounds (urban men born in 1956-65)

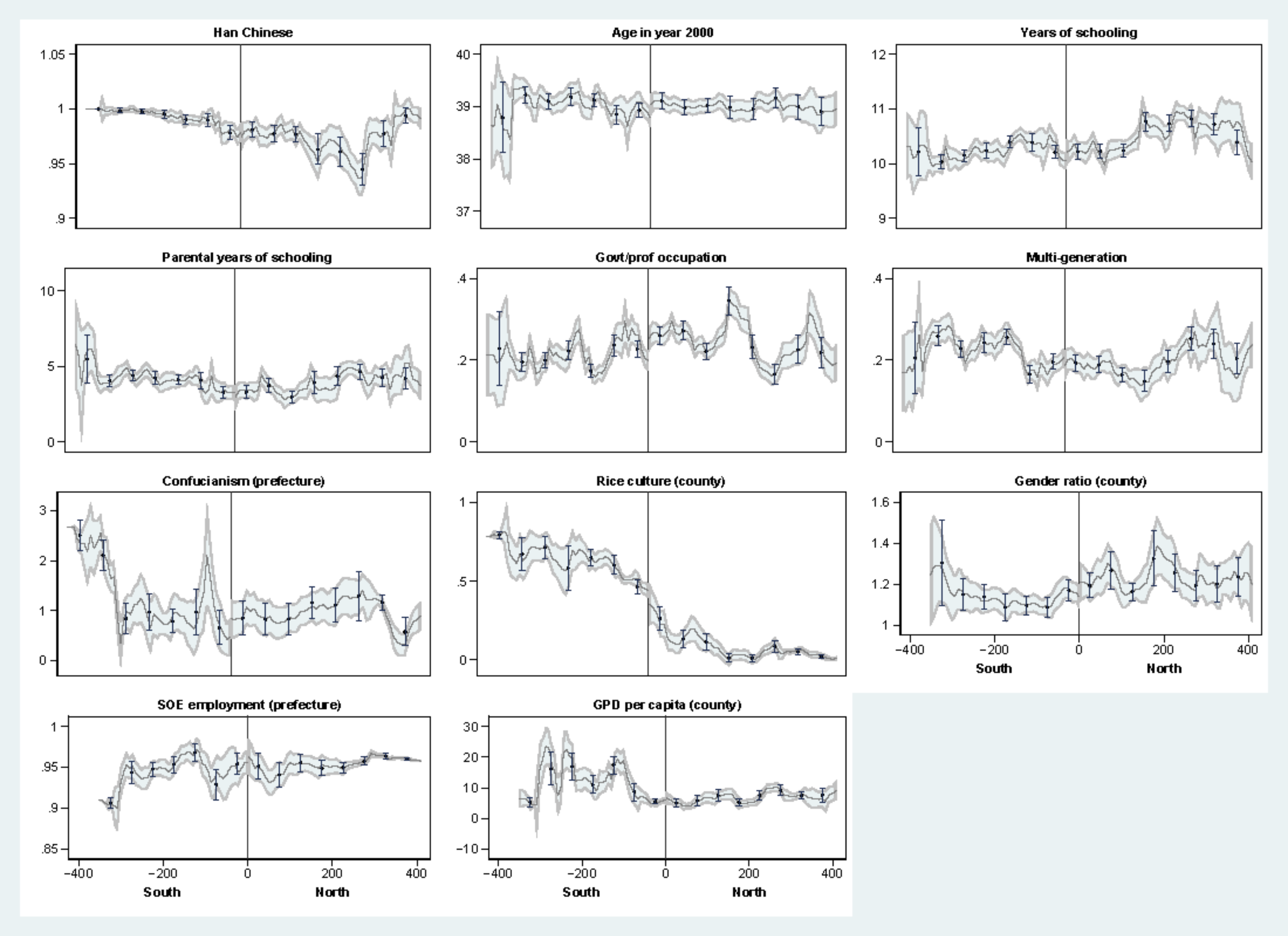

Notes: Sample comprises male residents with urban hukou born in 1956-65. Graphs depict fitted values from local polynomial estimate of the possible confounds (as labeled in each graph and explained in Table 2, Panel B) on distance from the Huai River, and corresponding 95 percent confidence intervals. Dots represent average of possible confounds within 50 km bins. All estimates are at individual level, except for rice culture, gender ratio, and GDP per capita by county, and Confucianism and SOE employment by prefecture.

Figure A2. Hypothetical policy boundaries

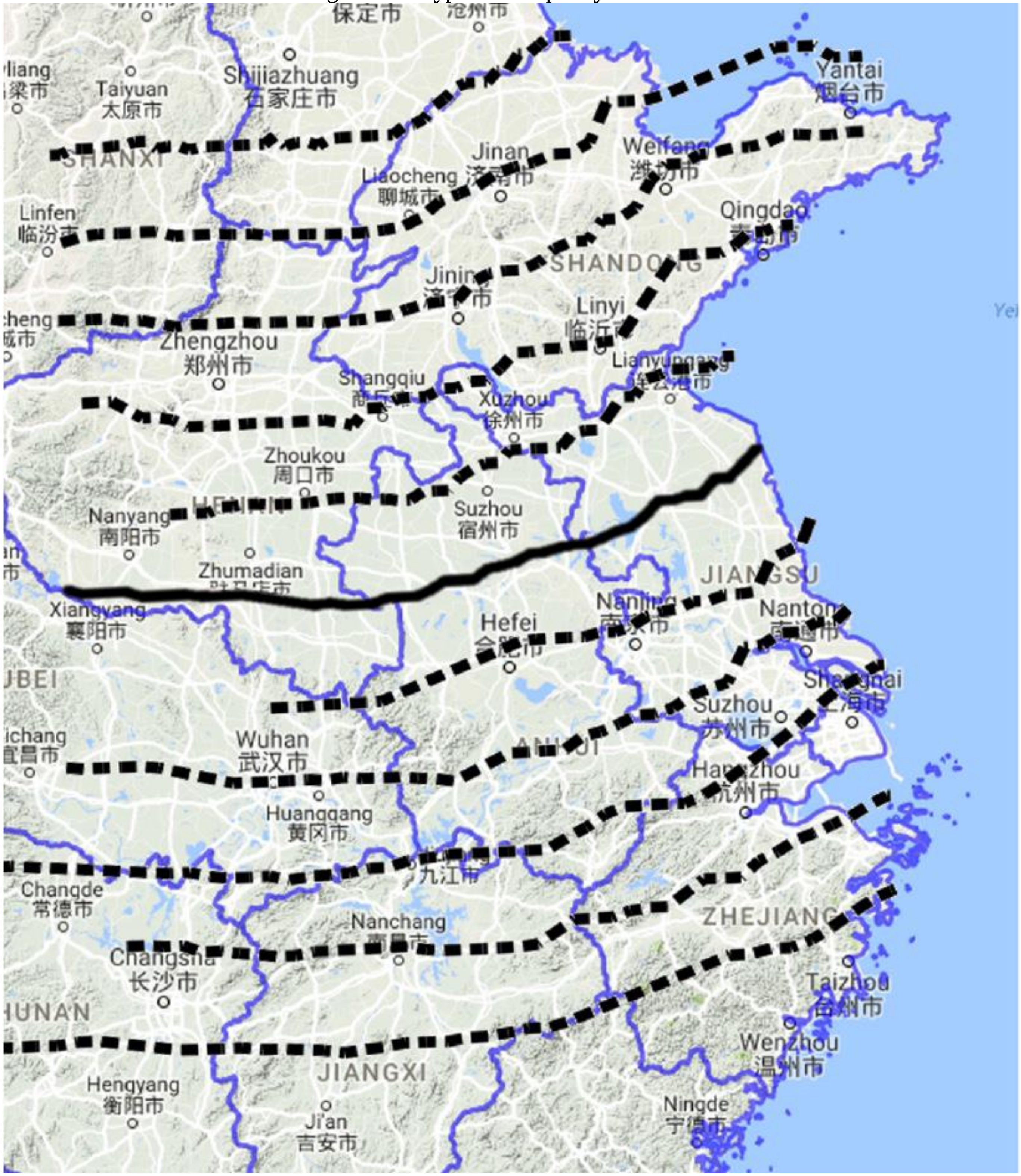

Notes: The solid line on the map is the Huai River and the black dotted lines are the hypothetical policy boundaries at 100-km intervals from 500 kms south to 500 kms north of the Huai River.

Figure A3. Regression discontinuity estimates with hypothetical policy boundaries

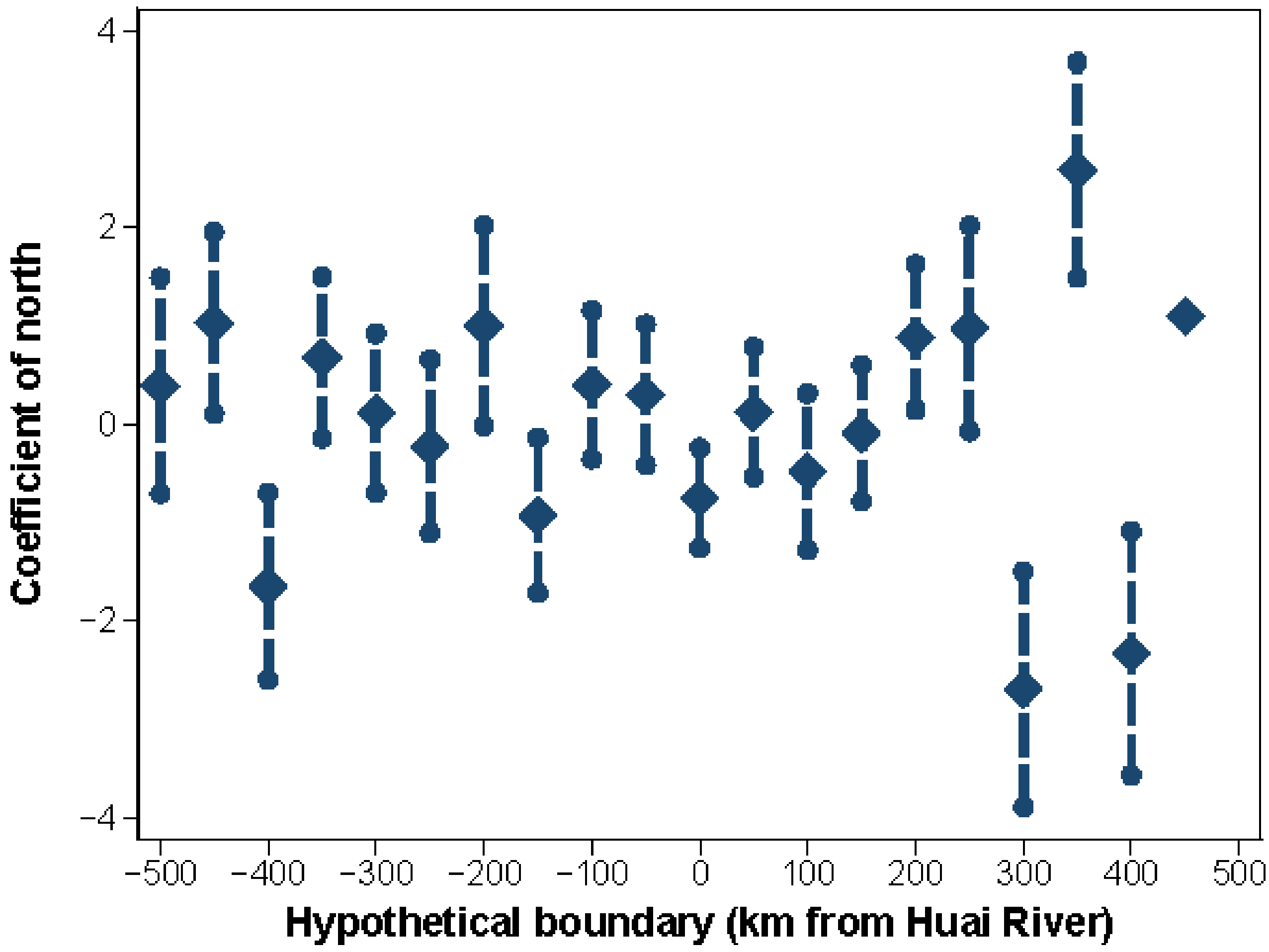

Notes: Coefficients of north and corresponding confidence intervals from GRD estimates with hypothetical policy boundaries at 50-km intervals from 500 kms south to 500 km north of the Huai River (Table A4).

Table A1. Additional institutional background statistics

A. Wages (Yuan per month)

	Henan, 1957				Anhui, 1959	Jiangsu, 1959
	Coal mining workers	Mechanical workers	Cigarette manufacturing workers	Coal staff	Coal mining	Mining
(a) Highest grade	105.60	94.50	71.25	175	105.6	105.62
(b) Lowest grade	28	29.50	26.50	36	26	33

Sources: Henan Provincial Gazetteer, Vol. 18 No. 25, Labor, Chapter 7.5, Tables 7-5-3, 7-5-4, 7-5-5, 7-5-7 (http://www.hnsqw.com.cn/sqsjk/hnsz/ldrsz/); Anhui Provincial Gazetteer, Labor, Chapter 5.1.2 (http://60.166.6.242:8080/was40/index_sz.jsp?rootid=15387&channelid=47966); Jiangsu Provincial Gazetteer, Labor Management, Chapter 6.5, (http://www.jssdfz.com/book/ldglz/DEFAULT.html.

B. Coal Subsidy

Heating coal, Harbin, 1975	20 Yuan per ton (Equivalent to US$13.30 at highly inflated official exchange rate)
Coal: International price	US$21.26 per ton
Average annual wage, 1978	615 Yuan

Sources: China Coal Association, Zhongguo Meitan Tongji Ziliao Huibian 1949-2004 (Collection of China Coal Statistics 1949-2004), Beijing: Meitan Gongye Chubanshe (Coal Industry Press), 2006; U.S. Energy Information Administration, https://www.eia.gov/totalenergy/data/annual/showtext.php?t=ptb0709; China Data Online.

Table A2. Data sources and construction

Variable	Construction/remarks	Source
Age at first marriage	Year of first marriage minus year of birth	1% sample of the China Population Census 2000
Early marriage	= 1 if men marry at or before 25 or women marry at or before 23; = 0 otherwise	
Male		
Han Chinese		
Age in year 2000	2000 minus year of birth	
Years of schooling	= 0 if illiterate; = 6 if primary school; = 9 if secondary school; = 12 if high school; = 14 if professional education; = 16 if undergraduate; = 18 if graduate	
Parental years of schooling	Average of parents' years of schooling	
Government official or professional occupation	= 1 if occupation code (GB/T6565-1999) starts with 1 or 2; = 0 otherwise	
Multi-generation	= 1 if live with (1) parents, (2) parents in law, (3) son and son-in-law, or (4) daughter and daughter-in-law; = 0 otherwise	
Gender ratio	Ratio of men over women	
Confucianism	Ratio of number of temples during the Ming and Qing dynasties to population of prefecture	Kung and Ma (2014); 1% sample of the China Population Census 2000
Rice culture	Percentage of cultivated area of prefecture or county sown with rice	Anhui, Henan and Jiangsu Statistical Yearbooks 1991; 1% sample of the China Population Census 2000
SOE (State-owned enterprise) employment	Ratio of employment in state-owned and collective enterprises to total employment by prefecture	
GDP (Gross domestic product) per capita	By prefecture for urban areas and by county for rural areas	China City Statistical Yearbook 2001; China Regional Economy Statistical Yearbook 2001
North		Google Maps
Distance to Huai River		
Average daily winter temperature, precipitation and sunshine hours	Daily average in December, January and February during 1956-87	China Meteorological Administration
Relative cohort loss rate	See Meng, et al. (2015) and Chu, et al. (2018)	1% sample of the China Population Census 2000
Education loss of sent down youth	High school graduation rate of cohort affected by the movement (born in 1946-62) minus that of those unaffected (born in 1963-80).	
Historical agricultural population	Percentage of county total population as agricultural in early 1930s	Xiao (1977)
Historical tenancy rate	Percentage of county agricultural population as tenants or semi-tenants in early 1930s	

Table A3. Urban men (born 1956-65): Falsification and robustness

Variables	Preferred estimate	Sample: Rural men	Counterfactual boundaries: Latitude 32.5°N	Counterfactual boundaries: North 50km	Counterfactual boundaries: South 50km	Linear dist. function
	(1)	(2)	(3)	(4)	(5)	(6)
North	-1.49***	-0.53	0.22	1.00*	0.53	-1.24***
	(0.40)	(0.32)	(0.51)	(0.51)	(1.10)	(0.35)
Observations	4,895	42,455	7,979	4,433	3,704	3,405
Counties	90	127	144	85	62	62
BIC	23,668	212,826	38,864	21,658	18,231	16,467
Bandwidth	82.46 km	114.72 km	0.93°	66.98 km	56.40 km	59.91 km
	Kernel: Triangular	Kernel: Epanech-nikov	Multi-level model	Sample: Urban couples	Sample: Incl. adjacent provinces	Sample: Incl. adjacent provinces
	(7)	(8)	(9)	(10)	(11)	(12)
North	-1.45***	-1.39***	-1.24***	-1.14***	-0.65**	-0.49**
	(0.39)	(0.38)	(0.31)	(0.37)	(0.30)	(0.20)
Observations	5,842	6,003	4,895	3,229	12,394	45,072
Counties	109	111	90	101	241	1,111
BIC	n.a.	n.a.	23,425	15,005	60,319	222,630
Bandwidth	99.51 km	101.51 km	82.46 km	92.27 km	175.90 km	n.a.

Notes: Estimated by OLS (except columns (6)-(7) by Stata routine, rdrobust), with quadratic distance polynomials and MSE-optimal bandwidth; Sample comprises married male residents in the 2000 census with urban hukou within MSE-optimal bandwidth, born in 1956-65, who did not move from neighborhood of birth; Dependent variable is age at first marriage; Standard errors clustered by county in parentheses (***$p < 0.01$, **$p < 0.05$, *$p < 0.1$). Column (1): Preferred estimate from Table 5, column (6); Column (2) repeats column (1) but replaces the urban sample by its rural counterpart; Column (3) specifies boundary as 32.5°N and assigns distance by latitude, with optimal bandwidth of 0.93°; Columns (4)-(5) specify boundary as 50 kms north or south of the Huai River; Column (6) repeats column (1) with linear distance polynomials; Columns (7)-(8) repeat column (1) with alternative density functions; Column (9) repeats column (1) using a multi-level model that includes county fixed effects, county-specific random coefficients for age, Han Chinese, education, occupation, living with parents, and province-specific coefficients for Confucianism, rice culture, gender ratio and GDP per capita; Column (10) excludes men whose wife holds rural hukou; Column (11) includes urban men in adjacent provinces (Shandong, Hebei, Shanxi, Hubei, Jiangxi, Zhejiang, and Shanghai); Column (l) repeats column (12) with parametric RD method.

Table A4. Counterfactual boundaries

Shift to south

	-50 km	-100 km	-150 km	-200 km	-250 km	-300 km	-350 km	-400 km	-450 km	-500 km
North	0.12	-0.48	-0.09	0.89**	0.97*	-2.69***	2.58***	-2.33***	1.11*	0.04
	(0.34)	(0.41)	(0.35)	(0.38)	(0.53)	(0.61)	(0.56)	(0.63)	(0.56)	(0.48)
Observations	20,920	16,268	13,591	17,909	14,826	18,114	12,460	8,779	8,913	5,830
Counties	384	283	228	300	244	328	236	182	199	172
BIC	101592	79137	65979	90103	74765	91453	63714	45979	46259	29602
Bandwidth	254.37	184.08	142.59	179.05	134.74	188.57	138.39	106.54	125.25	113.35

Shift to north

	50 km	100 km	150 km	200 km	250 km	300 km	350 km	400 km	450 km	500 km
North	0.30	0.40	-0.93**	1.00*	-0.22	0.11	0.68	-1.65***	1.03**	0.39
	(0.37)	(0.39)	(0.40)	(0.52)	(0.45)	(0.41)	(0.42)	(0.48)	(0.47)	(0.56)
Observations	12,324	10,071	8,277	11,660	13,386	10,963	10,489	5,554	4,098	5,050
Counties	243	209	188	287	351	305	319	185	164	192
BIC	60180	48978	40231	55921	64344	52506	50084	25923	19298	24441
Bandwidth	176.25	149.00	121.61	174.91	208.99	167.07	183.86	103.29	101.47	121.59

Notes: Estimated by OLS with quadratic distance polynomials and MSE-optimal bandwidth; Sample comprises married male residents in the 2000 census with urban hukou within MSE-optimal bandwidth of Jiangsu, Anhui, Henan, Hebei, Shanxi, Shanghai, Zhejiang, Fujian, Jiangxi, Shandong, Hubei, Hunan provinces, born in 1956-65, who did not move from neighborhood of birth; Dependent variable is age at first marriage; Standard errors clustered by county in parentheses (***p < 0.01, **p < 0.05, *p < 0.1).

Table A5. Regression discontinuity: Alternative methods and specifications

Variables	(1)	(2)	(3)	(4)	(5)	(6)
	Age at first marriage					Marry early
	All cohorts	1926-35	1936-45	1946-55	1956-65	1956-65
			A. Men: Parametric GRD			
North	-0.72***	0.34	0.51*	-0.86***	-	0.16***
					1.36***	
	(0.23)	(0.37)	(0.29)	(0.30)	(0.25)	(0.03)
Observations	43,761	4,988	8,213	13,155	17,405	17,668
Counties	372	367	372	371	372	372
BIC	229743	28,445	44,958	68,575	84,274	22,354
			B. Women: Parametric GRD			
North	-0.45**	-0.11	0.56*	-0.03	-	0.19***
					0.98***	
	(0.18)	(0.33)	(0.29)	(0.20)	(0.20)	(0.04)
Observations	27,860	2,591	4,211	8,087	12,971	13,041
Counties	372	338	362	370	372	372
BIC	140331	13,451	21,695	41,432	59,187	18,652
			C. Men: Non-parametric GRD with asymmetric bandwidth			
North	-1.07***	0.08	0.79*	-1.60***	-	0.16***
					1.31***	
	(0.40)	(0.52)	(0.47)	(0.48)	(0.39)	(0.05)
Observations	12,787	2,519	4,240	4,834	5,292	6,986
Counties	99	166	173	125	99	126
BIC	67,170	14,298	23,412	25,403	25,658	8,543.5
Bandwidth (km)	78.19/	145.30/	187.44/	91.19/	78.50/	115.78/
	99.08	139.64	122.49	136.00	100.77	101.48
			D. Women: Non-parametric GRD with asymmetric bandwidth			
North	-0.51	-0.35	0.45	-0.95**	-0.74**	0.14***
	(0.36)	(0.54)	(0.49)	(0.43)	(0.34)	(0.05)
Observations	18,836	2,014	3,625	6,499	9,025	5,882
Counties	145	135	151	169	166	140
BIC	99,576	11,431	19,934	34,352	44,165	8,311.2
Bandwidth (km)	148.00/	130.80/	116.38/	134.03/	170.15/	138.76/
	105.81	111.69	139.57	147.47	125.13	111.15

Notes: Panels A and B estimated by OLS, using parametric GRD with quadratic distance functions; Panels C and D estimated by Stata routine, rdrobust, using non-parametric GRD with quadratic distance functions and asymmetric MSE-optimal bandwidths (reported as "south/north"). Sample: men and women with urban hukou in all cohorts; Standard errors clustered by county in parentheses (***p < 0.01, **p < 0.05, *p < 0.1).

Table A6. Mechanism: Robustness

Variables	(1)	(2)	(3)	(4)	(5)	(6)
	Education		All counties	Govt/prof occupation		All counties
	Less	More		No	Yes	
North	-1.39***	-1.27***	-1.34***	-1.45***	-1.07***	-1.38***
	(0.46)	(0.43)	(0.26)	(0.40)	(0.58)	(0.25)
Education			0.12***			
			(0.02)			
North x education			0.04**			
			(0.02)			
Govt/prof occupation						-0.26***
						(0.09)
North x govt/prof occupation						0.14
						(0.12)
Observations	9,824	7,581	17,405	13,454	3,951	17,405
Counties	371	370	372	372	363	372
BIC	47,911	36,272	84,213	65,662	5,779	18,582

Notes: Estimated by OLS with quadratic distance functions. Sample comprises married male residents in the 2000 census from all 372 counties in Jiangsu, Anhui and Henan, with urban hukou, born in 1956-65, who did not move from neighborhood of birth; Dependent variable is age at first marriage; Standard errors clustered by county in parentheses (***p < 0.01, **p < 0.05, *p < 0.1). Columns (1)-(3): Estimates on individuals with below and above median years of education; Columns (4)-(6): Estimates on individuals in government or professional occupation or otherwise.

Appendix C: Differences in Differences

The GRD analyses estimate the local average treatment effect (LATE) of the heating policy in the neighborhood of the Huai River. Besides, it is also useful to understand the average treatment effect (ATE) of the heating policy, which is the effect on the broader population.

To investigate, we apply an empirical strategy of Differences in Differences (DID) and estimate a regression of the age at first marriage among all people with urban hukou:

$$a_j = \lambda_0 + \lambda_1 N_j + \sum_{t=1926/35}^{1956/65} \lambda_{2t} C_{jt} + \sum_{t=1926/35}^{1956/65} \lambda_{3t} N_j \cdot C_{jt} + X'_{ji}\beta + \varepsilon_{ji}, \tag{C-1}$$

where $C_{jt} = 1$ if individual j was born in cohort t, λ_0 is a constant, λ_1 represents the north-south difference in age at first marriage across all urban birth cohorts, λ_{2t} represents the common time trend for birth cohort t, and λ_{3t} represents the north-south difference in the time trend for cohort t, which is due to the heating policy, and ε_{ji} represents a random error term. The other variables and coefficients are as defined in (8) and (11).

Besides identifying the ATE rather than the LATE, the DID analysis is also helpful as it relies on different identification assumptions from the GRD analyses. DID analyses assume that the outcome of interest follows the same trend in both the control and treatment groups subject to a possible time invariant difference. Typically, this would be validated by showing a common pre-treatment trend. Unfortunately, our sample contains only one cohort (1926-35) which married before the heating policy, and so, we cannot test the existence of a common pre-treatment trend. Nevertheless, since counties in the north and south of the Huai River belong to the same provinces, and so, are subject to similar regulations, the common trend hypothesis is justifiable. By comparison, the GRD analyses rely on the assumption that the heating policy is the only factor that affects marriage which is discontinuous at the Huai River. Although we have checked the robustness of the GRD finding in various ways, it is useful to apply tests that rely on different assumptions.

As a preliminary, Table A7 reports the age at first marriage among men and women in the various birth cohorts with urban hukou resident in areas north and south of the Huai River.

Among all cohorts of men and all cohorts of women except those born between 1946-55, the age at first marriage is significantly lower in the north than south.

Table A8 reports estimates for men and women with urban hukou, and including controls, X_{ji}, comprising prefecture fixed effects and demographic, social, cultural, and other factors that affect marriage. The coefficient of north represents the north-south difference in the age at first marriage in the oldest cohort (1926-35). The coefficient of north for the later cohorts represents the difference in the north-south difference in the age at first marriage between the respective cohort and the oldest cohort.

Referring to Table A8, column (1), for men, the coefficient of north is not significant, while the coefficients of north for the 1946-55 and 1956-65 cohorts are negative and significant. The estimate suggests that, in the 1956-65 cohort, northerners married $0.36 + 0.06 = 0.42$ years or 5 months earlier than southerners.[1]

The estimate of the ATE is smaller than the corresponding GRD estimate of the LATE in Table 5, column (5). Apparently, the heating policy had a smaller effect on the age at first marriage in the north vis-à-vis south as a whole as compared with the immediate north vis-à-vis south of the Huai River. Referring to Figure 3(d), the age at first marriage increased with distance from the Huai River towards the south and north. The DID estimate suggests that, controlling for province and other controls, the north-south difference becomes smaller with distance from the Huai River.

[1]For women, the coefficient of north for the 1956-65 cohort is negative but imprecise. Combined with the coefficient of north for the 1926-35 cohort, the estimated north-south difference in the 1956-65 cohort is 0.43 years and significant. A puzzle is that the coefficient of north for the 1946-55 cohort is positive.

Table A7. Urban residents: Age at first marriage

Variables	(1)	(2)	(3)	(4)	(5)	(6)	(7)
	Age at first marriage					% Early marriage	Marriage rate (%)
	All	1926-35	1936-45	1946-55	1956-65		
			Men				
North	23.58	22.49	23.06	24.01	23.87	75.87	99.23
	(3.29)	(4.16)	(3.55)	(3.20)	(2.74)	(42.78)	(8.76)
	[21,423]	[2,672]	[4,178]	[6341]	[8,232]	[21,590]	[21,590]
South	24.88	23.42	24.31	25.49	25.04	60.03	98.38
	(3.40)	(4.20)	(3.94)	(3.37)	(2.73)	(48.99)	(12.61)
	[22,338]	[2,316]	[4,035]	[6,814]	[9,173]	[22,705]	[22,705]
Total	24.24	22.92	23.67	24.78	24.49	67.75	98.79
	(3.41)	(4.20)	(3.80)	(3.37)	(2.80)	(46,74)	(10.91)
	[43,761]	[4,988]	[8,213]	[13,155]	[17,405]	[44,295]	[44,295]
North-south difference	-1.30	-0.93	-1.26	-1.48	-1.17	15.85	0.84
t-statistic	-40.65	-7.87	-15.19	-25.84	-28.30	36.19	8.13
			Women				
North	22.63	20.25	21.13	23.51	23.05	63.13	99.70
	(2.95)	(2.87)	(3.03)	(3.12)	(2.36)	(48.25)	(5.50)
	[11,507]	[1,055]	[1,740]	[3,205]	[5,507]	[11,542]	[11,542]
South	22.86	20.71	21.40	23.31	23.50	58.32	99.55
	(3.04)	(3.44)	(3.28)	(3.14)	(2.38)	(49.30)	(6.70)
	[16,353]	[1,536]	[2,471]	[4,882]	[7,464]	[16,427]	[16,427]
Total	22.77	20.52	21.29	23.39	23.31	60.31	99.61
	(3.01)	(3.23)	(3.18)	(3.13)	(2.39)	(48.93)	(6.23)
	[27,860]	[2,591]	[4,211]	[8,087]	[12,971]	[27,969]	[27,969]
North-south difference	-0.23	-0.46	-0.27	0.20	-0.45	4.81	0.15
t-statistic	-6.34	-3.58	-2.68	2.85	-10.69	8.11	1.95

Notes: Sample comprises residents in the 2000 census in Henan, Anhui, and Jiangsu born in 1926-65, with urban hukou, who did not move from neighborhood of birth. Upper rows report mean values, standard deviations in parentheses and frequencies in brackets; Lower rows report difference in means between north and south with t-statistics (***p < 0.01, **p < 0.05, *p < 0.1).

Table A8. Urban residents: Difference in differences

Variables	Men (1)	Women (2)
Constant	22.85***	19.97***
	(0.25)	(0.20)
Born 1936-45	0.85***	0.17
	(0.12)	(0.12)
Born 1946-55	1.86***	1.64***
	(0.10)	(0.11)
Born 1956-65	1.29***	1.39***
	(0.10)	(0.11)
North	-0.06	-0.26
	(0.20)	(0.18)
North x Born 1936-45	-0.33**	0.12
	(0.15)	(0.16)
North x Born 1946-55	-0.57***	0.48***
	(0.15)	(0.15)
North x Born 1956-65	-0.36**	-0.17
	(0.15)	(0.15)
Prefecture fixed effects	Yes	Yes
Covariates	Yes	Yes
Observations	43,572	27,860
Counties	372	372
R-squared	0.11	0.19
North-south difference		
Cohort 1936-45	-0.389**	-0.137
p-value	[0.035]	[0.419]
Cohort 1946-55	-0.624***	0.228*
p-value	[0.000]	[0.094]
Cohort 1956-65	-0.414**	-0.429***
p-value	[0.015]	[0.002]

Notes: Sample comprises residents in 2000 census with urban hukou born in 1926-65 who did not move from neighborhood of birth; Estimated by OLS with prefecture fixed effects and covariates (as listed in Table 5); Dependent variable: age at first marriage; Column (1): Men; Column (2): Women. Standard error clustered by county in parentheses (***p < 0.01, **p < 0.05, *p < 0.1). North-south difference is the sum of the coefficient of North and the coefficient of the interaction of North with the respective birth cohort.
